# Complete Heart Block and Ventricular Asystole Caused by Vagus Nerve Stimulation Therapy

**DOI:** 10.7759/cureus.53314

**Published:** 2024-01-31

**Authors:** Jarrod Warnock, Cody Ashcroft, Raphael J Sabado, Andrea Keithler, Serafim Perdikis

**Affiliations:** 1 Internal Medicine, Brooke Army Medical Center, San Antonio, USA; 2 Cardiology, Brooke Army Medical Center, San Antonio, USA

**Keywords:** vagus nerve stimulation, bradyarrhythmia, refractory epilepsy, transient ventricular asystole, complete heart block

## Abstract

Left vagus nerve stimulation (VNS) is an advanced therapeutic option for refractory, drug-resistant epilepsy. A 45-year-old woman with a history of refractory catamenial focal epilepsy since age 16, treated with a five-drug antiepileptic regimen and VNS (implanted eight and one-half years prior), presented with dyspnea, chest discomfort, and lightheadedness. During observation, symptoms recurred and were associated with bradycardia (<20 bpm) and a complete atrioventricular node (AVN) block. Following admission, she continued to experience recurrent symptomatic AVN block and transient ventricular asystole, temporally correlated with her baseline seizure activity and resultant activation of her VNS. Deactivation of VNS resolved her bradyarrhythmia, and she experienced no recurrence over 14 months of follow-up. This case highlights a therapeutic dilemma in cases of refractory epilepsy, with limited therapeutic options if seizure activity requires VNS to be controlled.

## Introduction

Vagus nerve stimulation (VNS) therapy is a common second-line non-pharmacologic therapy that has proven efficacy for the treatment of anti-epileptic drug-refractory epilepsy [1,2]. The process of placing a VNS stimulator involves positioning a lead wire at the cervical portion of the trunk of the left vagus nerve above the clavicle and secondary placement of a generator in the upper chest [1]. The device then delivers recurrent stimulation to the vagus nerve, either on a programmed time interval or, with newer adaptations, an automatic stimulation can be triggered by epileptic activity [1].

The relative safety of VNS therapy is also well defined, with minimal side effects mostly involving the perioperative period and relatively rare major adverse events that require the removal or deactivation of the device [2,3]. The most common side effects are divided into early complications related to surgical implantation itself and late complications related to the device and its mechanism. The early complications include intraoperative bradycardia and asystole, infection, hematoma, and injury to the vagus nerve causing hoarseness, dysphagia, or dyspnea due to vocal cord dysfunction. The late complications include late-onset arrhythmias, hoarseness, dyspnea and cough, the development of obstructive sleep apnea, overstimulation of the phrenic nerve, tonsillar pain, and vocal cord damage [4].

One serious complication mentioned above that would prompt removal, deactivation, or modification of the device is the occurrence of clinically significant bradycardia. While the incidence of intraoperative bradycardia with transient asystole is only 0.1% during implantation, late-onset atrioventricular node (AVN) block occurring after initial implantation is exceptionally rare, with only eight other known occurrences [5-12]. Herein we describe a case of a rare but severe side effect of VNS therapy causing late-onset complete heart block with transient ventricular asystole requiring deactivation of the device.

This article was previously presented as a meeting abstract at the 2022 Triservice ACP Meeting on September 8, 2022.

## Case presentation

A 45-year-old female patient with a history of refractory catamenial focal epilepsy since age 16 presented to the emergency department with worsening dyspnea and lightheadedness. Her seizures were uncontrolled on a five-drug antiepileptic regimen that included perampanel, levetiracetam, valproate, clobazam, and topiramate. She denied any medication non-compliance. A VNS was implanted eight and one-half years before presentation, with the generator replaced and upgraded two years ago. The device was interrogated during her inpatient admission and is included below in addition to the most recently available interrogation report. Device reports from an outside provider were not obtained during the patient's inpatient stay and were not available upon retrospective review. There was a device settings change made between admission and the most recent device report, but this report was unavailable in our records. However, the device reports were relatively unchanged in the reported metrics. The device settings reported during the inpatient stay were as follows: normal output current is 2.25 mA, frequency 20 Hz, pulse width 250 microseconds, on time 30 seconds, off time 1.1 min, duty cycle 35%, auto-stim settings of output 2 mA, pulse width 250 microseconds, on time 30 seconds. Magnet settings of output 2.5 mA, magnet on time 60 seconds, and magnet pulse width 250 microseconds. The last available device settings on record review dated June 19, 2020, were as follows: output current 2.25 mA, signal frequency 20 Hz, pulse width 250 microseconds, signal on time 30 seconds, signal off time 1.1 minutes, magnet current 2.50 mA, magnet pulse width 250 microseconds, magnet on time 60 seconds, auto-stim output current 2 mA, auto-stim pulse width 250 microseconds, auto-stim on time 30 seconds.

She was noted to have a prolonged QTc on presentation (616 ms) with tachycardia and associated hypokalemia (2.9 mEq/L), which was quickly corrected with normalization of the QTc interval. However, during observation, the patient had a witnessed episode of her index seizure symptoms, which were described as a staring spell with arm extension, followed by an episode of bradycardia (<20 bpm) due to a complete atrioventricular (AV) block. This was associated with the reproduction of her presenting symptoms of chest discomfort and lightheadedness, resulting in her admission to the cardiac care unit. Initial labs showed no evidence of a thyroid disorder, cardiac ischemia, infection, or infiltrative disorder. Her other prescription medications included Depo-Provera (Pfizer Inc., New York, United States) injection monthly, fluticasone propionate nasal spray, azelastine nasal spray benzonatate, and pramipexole 0.25mg daily. Over-the-counter medications included, as needed, Mucinex DM (Reckitt Benckiser Pharmaceuticals Inc., Slough, United Kingdom), fish oil (1 g), and a multivitamin daily. No AV nodal-blocking agents were identified during medication reconciliation.

**Figure 1:**
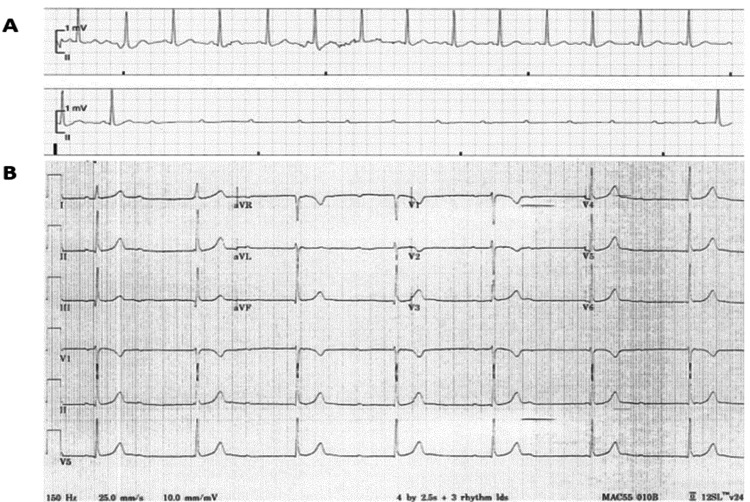
Complete heart block during vagus nerve stimulation A) Telemetry tracing demonstrating high-degree atrioventricular (AV) block with ventricular asystole lasting for nine seconds. B) A 12-lead ECG showing complete atrioventricular block (CAVB).

During her hospital stay, the patient had two separate episodes of her baseline seizure activity requiring treatment by her VNS. These episodes were temporally correlated with symptomatic complete heart block and transient ventricular asystole (Figure 1) observed on continuous telemetry monitoring. A transthoracic echocardiogram showed normal ventricular function without structural abnormalities.

Neurology interrogated her VNS and confirmed a temporal correlation between seizure activity leading to increased VNS activity and subsequently her bradyarrhythmia. The VNS was deactivated with no recurrence of her symptoms or arrhythmia during her subsequent hospital course. Post-discharge ambulatory cardiac monitoring for 14 days showed no additional episodes of high-grade AV nodal block. Her antiepileptic medications and birth control were also adjusted, resulting in no reported increase in her epileptic activity. During follow-up over the following 14 months, the patient reported no return of her symptoms of chest discomfort and lightheadedness.

## Discussion

VNS therapy involves the surgical implantation of an electrical device under the skin of the anterior chest with a lead, which is then tracked and wound around the vagus nerve. The device typically has a baseline level of stimulation, and newer devices (such as the device to which the patient was upgraded two years prior to presentation) have an auto-stimulation feature that, upon detecting early seizure activity, will increase output [13].

Notably, after both the device upgrade two years prior to presentation and after an adjustment to the device settings three months prior to presentation, the patient reported an increase in her seizure burden. Additionally, after reviewing available records from June 2020 and comparing them to settings obtained during inpatient admission, there was no significant change in settings. However, she had not experienced the symptoms that brought her to the emergency department, despite the increase in seizure burden and presumed increase in auto-stim functions. Regardless, the temporal correlation of the patient’s recurrent symptomatic AVN block and transient ventricular asystole with increased VNS activity was indisputable on device interrogation by neurology, and deactivation resulted in the resolution of her symptoms.

As late-onset complete heart block with transient ventricular asystole is an exceedingly rare complication attributed to VNS therapy, it was essential to perform a thorough evaluation of the patient to rule out more likely causes and to ensure that her pre-syncopal symptoms were not improperly attributed to increased seizure burden, as can sometimes occur. Medication side effects, electrolyte abnormalities, thyroid disorders, ischemic heart disease, cardiomyopathy, infiltrative disorders, infection, and structural heart disease were all considered. Laboratory evaluation and cardiac imaging were performed consistent with this differential, and all results were unremarkable, making the aforementioned differentials less likely. Additionally, extended ambulatory cardiac monitoring after discontinuation of VNS therapy showed no recurrence of her AV block and was crucial to confirm the complete resolution of her ECG abnormalities and the absence of any baseline electrophysiologic abnormalities.

To our knowledge, late-onset complete heart block with transient ventricular asystole attributed to VNS therapy has only previously been reported in eight other studies, as electrophysiologic side-effects usually manifest intraoperatively (only in 0.1%), if at all [5-12]. A majority (five out of eight) of the previous cases noted the onset of symptomatic AVN blockade, requiring discontinuation of VNS therapy within three years of placement. However, the case described above is one of only four cases that showed this side effect more than three years out from placement, with the previous two cases describing complete heart block with ventricular asystole nine years and thirteen years after initial VNS implantation [5,11]. Our patient experienced this complication eight and one-half years after initial implantation.

The exact mechanism that led to the bradyarrhythmia in this patient (and other patients with this complication during and after implantation) is uncertain. ­­One proposal suggests VNS directly increases parasympathetic activity on the AVN through anatomic vagus nerve variations or inadvertent electrode placement, although this would have been unlikely with the remote placement of this patient's VNS [7,14]. Another proposal posits that aberrant neurological pathways play a role, although this was suggested in the setting of three patients with preexisting neurological diseases other than epilepsy [14]. Finally, one study using fMRI possibly supports a mechanism that centers around long-term changes and central nervous system remodeling due to VNS and its effects on the nucleus tractus solitarius and its effects on autonomic functions in the hypothalamus and insular cortex, which in the setting of this patient seems the most likely [15].

## Conclusions

The case presented above is unique in that the development of symptomatic, complete AVN dissociation with transient ventricular asystole is exceedingly rare outside of the intraoperative setting of VNS therapy. Because the differential for the above case of presenting symptoms most consistent with syncope is broad and this side effect is so uncommon, it is important for physicians to consider this rare side effect of VNS therapy, as there is a high risk that it may be overlooked and syncopal symptoms misattributed to an incorrect alternative etiology. This case adds to the previously reported cases to confirm and support complete heart block and transient ventricular asystole as a known, albeit rare, complication of VNS therapy.
